# ^18^F-FDG PET-CT incidental lung findings in asymptomatic COVID-19 patients: evidences from the Italian core of the first pandemic peak

**DOI:** 10.22038/AOJNMB.2021.58035.1405

**Published:** 2022

**Authors:** Bonanomi Alice, Bonaffini Pietro Andrea, Spallino Marianna, Dulcetta Ludovico, Franco Paolo Niccolò, Valle Clarissa, Marra Paolo, Bruno Andrea, Sironi Sandro

**Affiliations:** 1 Department of Radiology, Papa Giovanni XXIII Hospital, Bergamo, Italy; 2 School of Medicine, University of Milano Bicocca, Milano, Italy; 3 Department of Nuclear Medicine, Papa Giovanni XXIII Hospital, Bergamo, Italy

**Keywords:** COVID-19, Interstitial pneumonia, Asymptomatic, PET-CT, FDG uptake

## Abstract

**Objective(s):**

To illustrate incidental ^18^F-FDG PET-CT findings and related CT alterations of suspicious pulmonary interstitial involvement in asymptomatic oncologic patients during the first COVID-19 outbreak in the core of Italian peak.

**Methods:**

We retrospectively evaluated the ^18^F-FDG PET-CT follow-up examinations performed during the first Italian COVID-19 outbreak (March 3rd-April 15th, 2020) in 10 asymptomatic oncologic patients with a highly suspicious interstitial pulmonary involvement on CT. Six cases were confirmed SARS-CoV-2 by molecular tests. The following parameters were assessed: 1) lung involvement on co-registration CT as extension (laterality, number of lobes), pattern (ground-glass opacities/GGO, consolidations, mixed) and stage (early, progressive, peak, and absorption); 2) the maximum standardized uptake value (SUV_max_) of lung lesions on ^18^F-FDG PET.

**Results:**

The involved lobes were 5 in 5 cases (3 confirmed SARS-CoV-2), 2-4 in 4 cases and 1 in 1 case. GGO were found in all patients; 3 cases also showed a combination of GGO and peripheral consolidations (mixed). Five cases were suggestive for an early stage of interstitial pneumonia, 4 for progressive and 1 for peak. All the lung lesions showed increased FDG uptake. In early stages SUV_max _ranged from 1.5 to 11, in progressive from 3.3 to 6.8, in peak from 2.4 to 7.7. SUV_max_ ranged 1.5-11 in patients with only GGO and 2.8-7.7 in those with mixed pattern.

**Conclusions:**

^18^F-FDG PET-CT findings in suspected COVID-19 pulmonary involvement of asymptomatic oncologic patients showed an increase in FDG uptake of GGO and consolidations, but with a wide and apparently nonspecific range of SUV_max_ values.

## Introduction

 Coronavirus disease 2019 (COVID-19), caused by SARS-CoV-2 (Severe Acute Respiratory Syndrome Coronavirus 2) infection, mainly affects the respiratory system, leading to several flu-like symptoms (weakness, cough and fever).

 In more severe cases, COVID-19 evolves in bilateral interstitial pneumonia, which can precipitate in acute respiratory distress syndrome (ARDS). However, in many cases COVID-19 patients can be completely asymptomatic. According to the literature, the prevalence of asymptomatic cases has been described as a large part of all COVID-19 infections, ranging from 30.8% to 73% (1, 2). Consequently, it is expected that undocumented and asymptomatic patients could represent an important source for SARS-CoV-2 spread (3). 

 Computed tomography (CT) represents the imaging mainstay in the assessment of lung involvement in COVID-19 patients, being able to accurately depict the extent of disease and its evolution. CT also demonstrated higher sensitivity, compared to chest X-ray (CXR), in the detection of early lung disease stages, even in asymptomatic patients. Particularly, it has been described that more than 50% of asymptomatic infected patients show lung abnormalities on CT investigations, especially ground glass opacities (GGO)(2).

 Fluorine 18 fluorodeoxyglucose (^18^F-FDG) PET-CT is known to identify, beside neoplastic processes, inflammatory and infective tissues, due to their increased glycolytic metabolism. Therefore, not surprisingly ^18^F-FDG PET-CT can demonstrate increased metabolic activity also in cases of interstitial pneumonia, although it does not play a specific role per se in COVID-19 imaging workup (4-6). Furthermore, as functional imaging techniques, nuclear medicine studies could theoretically identify metabolic active sites of inflammation in the early stages (7, 8).

 On these bases, the aim of this study is to describe the main incidental ^18^F-FDG PET-CT findings and related CT alterations of suspicious pulmonary interstitial involvement in asymptomatic oncologic patients during the first COVID-19 outbreak in the core of Italian peak.

## METHODS

***Study population***

 We retrospectively enrolled all patients with the following inclusion criteria: a) ^18^F-FDG PET-CT examinations performed for oncologic follow-up purposes during the first Italian COVID-19 outbreak (March 3rd-April 15th, 2020); b) incidental detection of highly suspicious SARS-CoV-2 related pulmonary involvement, not ascribable to the underlying neoplastic pathology on co-registration CT; c) completely asymptomatic patients at the time of PET-CT scans (except for one patient who referred an episode of fever 37.5°C, the days before the exam).

 Patients’ medical history and outcome were retrospectively retrieved from electronic medical records (Table 1). Particularly, results of real-time reverse polymerase chain reaction (RT-PCR) from pharyngeal swabs and/or antibodies from serological analysis were assessed. If not available, patients were considered in the analysis, whenever presenting at CT a pattern of pulmonary involvement highly suspicious for interstitial pneumonia (also considering the epidemiological contingency) (9). For those patients who performed further imaging studies during follow up, they were also retrospectively reviewed from the picture archiving and communication system.

**Table 1:** Demographic characteristics of included patients. #all PET were performed for follow-up

**# Patient**	**Sex**	**Age**	**PET-CT Indication#**	**Suspect** **COVID-related symptoms**	**Primary** **tumor Uptake**	**Distant** **tumor Uptake**	**SARS-CoV-2 diagnosis**
**001**	F	57	Nasopharynx cancer	None	None	None	Positive quantitative serological test
**002**	M	71	Hepatocarcinoma	None	None	Pulmonary nodule	Suggestive CT (lung)
**003**	M	57	Oropharynx cancer	None	Yes	None	Positive quantitative serological test
**004**	M	69	Melanoma	None	Yes	Axillary Lymph node	Suggestive CT (lung)
**005**	F	50	Breast cancer	None	Yes	Axillary Lymph node	Suggestive CT (lung)
**006**	F	71	Breast cancer	None	Yes	None	Positive nasal swab (PCR)
**007**	M	72	Duodenal cancer	None	Yes	None	Positive nasal swab (PCR)
**008**	F	77	Adnexal cancer	Fever the day before	Yes	None	Positive nasal swab (PCR)
**009**	F	81	Urothelial cancer	None	Yes	None	Suggestive CT (lung)
**010**	M	69	Lymphoma	None	No	None	Positive nasal swab (PCR)

^18^ ***F-FDG PET-CT and CT imaging: Scanning protocol***

 According to EANM guidelines (10) ^18^F -FDG PET-CT was performed after >4 hours fasting and with blood glucose level <200 mg/dL. We administered an intravenously dose of 3.7 MBq/kg and performed imaging 55-75 min post glucose injection. Total-body PET images were acquired in 3D with Flow Motion technique (Biograph mCT Flow S64 4R, Siemens) at a speed of 1.1 mm/sec (200×200 matrix) corresponding to a stop and go acquisition of 2.00 min per bed. Low dose CT was recorded for the correction of attenuation and anatomical localization, with the following parameters: 120 kV, CARE Dose4D (performs automatic tube-current modulation in the angular and longitudinal directions and also adapts to different anatomical regions and patient sizes), 0.5 sec per rotation, delay 2 sec, pitch 1.5, layer thickness 3 mm, 512×512 matrix and a pixel reconstruction of 0.98 mm.

 Before PET completion, total body CT scans were acquired in the same session, with patient supine, 100 kV and automated tube current modulation. Collimation, pitch and rotation time were 16×1.2 mm, 1.1:1 and 0.5 sec, respectively. Slice thickness was 2.0 mm. All CT studies were performed unenhanced. Moreover, 4 patients additionally underwent contrast enhanced CT (CECT) studies: 2 patients 30 days after the pneumonia evidence and 2 one week before the PET-CT. All the 4 CECT scans were performed as standard oncologic follow-up, on a 64-detector CT scanner (Brilliance, Philips) during the intravenous bolus injection of 1.3-1.5 mL/kg of non-ionic contrast medium (iomeprol 350 mg/mL; Bracco Imaging), at flow rate of 2.5-3.5 mL/s and followed by a rapid saline solution flush (30-40 mL, flow rate 3 mL/s). Images were acquired in a single portal venous phase, about 80-90 seconds after contrast injection.

***Images analysis***

 Image analysis for CT was performed independently by two radiologists, with 3 years of experience in CT imaging (A.B. and P.N.F.). Unenhanced co-registration CT of PET scans was used for lung assessment and the following characteristics were recorded: a) extension of lung involvement, in terms of laterality (unilateral/bilateral) and number of involved lobes (from 1 to 5); b) pattern, as GGO, consolidations or mixed (both GGO and consolidations); c) stage, which included early, progressive, peak and absorption, as described by Feng P et al (11). In the mentioned study, stages are defined from the day of onset of symptoms. Being our patients asymptomatic, in this study stages were assigned according to morphologic appearance of lung alterations on CT. Namely, early stage (stage 1) is characterized by GGO, mostly subpleural at lower lobes. In the progressive stage (stage 2) there is a bilateral extension of the pneumonia, with diffuse GGO, crazy paving pattern and initial consolidations. The peak stage (stage 3) is characterized by prevalent parenchymal consolidations with residual parenchymal bands and occasionally GGO. The absorption stage (stage 4) represents a gradual resolution.

 PET-CT images were analyzed independently by two nuclear physicians with more than 5 years of experience in PET-CT imaging (A.B. and M.S.). All the FDG avid foci were qualitatively evaluated, and maximum standardized uptake value (SUV_max_) was measured. We recorded the SUV_max_ value of the most FDG-avid lung abnormality.

***Statistical analysis ***

 Continuous data are expressed as mean ± standard deviation and categorical data as percentage. Descriptive statistics were calculated with Excel software (Microsoft Office 2016).

## RESULTS

 The final population study included 10 patients (5 females; mean age 67 years, range 50-81), of whom 6 were subsequently confirmed positive for SARS-CoV-2 infection: 4 with molecular swab and 2 with quantitative serological test. The remaining 4 cases, although untested, had CT findings strongly consistent with COVID-19-related interstitial pneumonia.

 In terms of distribution of lung involvement on CT, in most cases lung abnormalities were multiple and bilateral: 5 patients had involvement of all the pulmonary lobes and 4 patients had 2-4 affected lobes. One patient had a single affected lobe.

**Figure 1:**
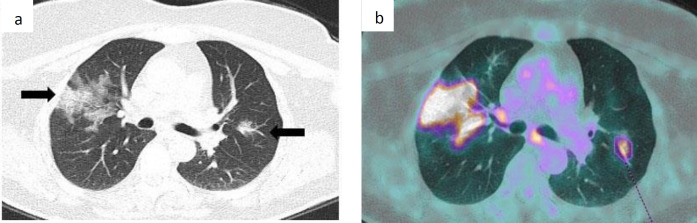
CT (**a**) and PET-CT (**b**) images from a 57 years old female in semestral follow-up for a nasopharyngeal cancer and asymptomatic at the moment of the acquisition. In the co-registration CT (**a**) there are bilateral GGO in the right and left upper lobes (black arrows); no consolidation are noted, resulting in an early stage. These GGO demonstrate intense FDG uptake (**b**), with SUV_max_ in the right lobe of 11.02 and SUV_max_ in the left lobe of 6, 72. Otherwise, PET was negative

**Figure 2:**
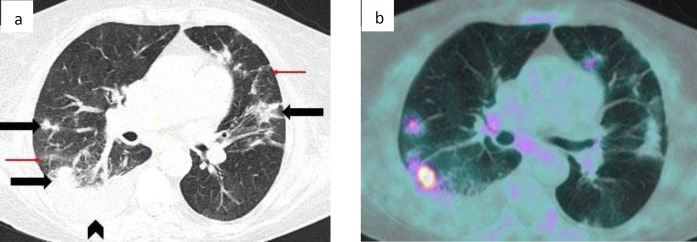
Female, 72 years old, with urothelial cancer. Axial CT image (**a**) shows bilateral compact consolidations (black arrows), mixed with peripheral GGO and reticulation (red arrows) and right pleural effusion (black arrowhead); findings consistent with a peak stage. On corresponding PET scan (**b**) SUV_max_ of the nodular consolidation adjacent to the pleural effusion was 7.7. The patient was a poorly compliant patient, but the family members did not report any symptom in the days before the scan

 The FDG uptake intensity was extremely variable in our population (Table 2). SUV_max_ in patients with 2-4 affected lobes ranged from 2.7 to 11, with a mean value of 6.8. In patients with 5 affected lobes SUV_max_ ranged from 1.5 to 6, with a mean value of 3.8. The patient with 1 involved lobe had a SUV_max_ of 9.4. According to mentioned CT stages, we found an overlap in SUV_max _values. In early stage SUV_max_ ranged from 1.5 to 11, with a mean SUV_max_ of 6.3 (Figure 1); in progressive stage SUV_max_ ranged from 3.3 to 6.8, with a mean SUV_max_ of 5.1 and in the single patient in the peak stage SUV_max_ ranged from 2.4 to 7.7, with a mean SUV_max_ of 5.1 (Figure 2). None of the patients was in the absorption stage.

**Table 2:** SUV_max_ values according to extension, stage and pattern of lung abnormalities as revealed by co-registration CT.* Feng Pan, Tianhe Ye et al, “Time Course of Lung Changes at Chest CT during Recovery from Coronavirus Disease 2019”. SUV_max_: maximum standardized uptake value; GGO: ground glass opacities

**Extension (N° lobes involved)**	**N° of patients (total 10)**	**SUV**_max_ **(range)**	**SUV**_max_ **(mean)**
**All the lobes**	5	1.5 - 6	3.8
2-4 lobes	4	2.7 - 11	6.8
1 lobe	1	9.4	9.4
Stage*			
1 - early	5	1.5 - 11	6.3
2 - progressive	4	3.3 - 6.8	5.1
3 - peak	1	2.4 - 7.7	5.1
4 - absorption	0	0	0
**Pattern**			
GGO	10	1.5 - 11	6.3
Consolidation	0	0	0
Mixed (GGO + consolidation)	3	2.8 - 7.7	5.2

 All the patients were asymptomatic for any potential lung involvement at the time of PET-CT, except for one patient who had a single episode of mild fever (37.5°C) the day before the exam.

 Due to the emergency situation and the lack of symptoms, the patients were discharged and entrusted to the family doctor, in quarantine. 

 During follow-up, none of the patients developed symptoms nor required hospitalization. Two patients (one tested positive for SARS-CoV2 with molecular swab), underwent CECT 30 days after the first PET-CT, showing an improvement of lung involvement.

## Discussion

 In line with the increasing pandemic emergency during the first COVID-19 outbreak, nuclear medicine imaging workflow at our Institution had been reduced but not completely interrupted. This was aimed to guarantee oncologic monitoring as largely as possible, leading consequently to the occasional incidental detection of pulmonary findings consistent with interstitial pneumonia during routine follow up ^18^F-FDG PET-CT scans in oncologic patients. 

 Considering the concomitant epidemiological setting and the related CT pattern, lung findings were highly suspicious at first sight and suggestive of COVID-19 related pneumonia. A 60% of these cases were then confirmed positive with molecular or serological test. Notably, all the patients were completely asymptomatic for lung involvement at the time of the examination and none of them developed pulmonary symptoms or symptoms requiring hospitalization during follow-up.

 Many studies showed that asymptomatic infected individuals can be a contagious source of SARS-CoV-2. Moreover, a significative percentage of these patients could rapidly progress to symptomatic disease (3, 12). Therefore, it is important for epidemiologic and clinical issues to identify, isolate and monitor also asymptomatic cases. According to several studies, the percentage of asymptomatic COVID-19 patients is deeply variable depending on the examined population. Generally, asymptomatic cases seem to account for about 40-45% of total cases of infections and may be associated with subclinical lung abnormalities detected by CT (13, 14). As reported by Varble N. et al, 65% of initially asymptomatic patients presented lung infiltrates at CT scans and the most common pattern was GGO (94%) followed by consolidations (46%). Only half of these asymptomatic patients with pulmonary disease would develop symptoms 1 to 5 days after initial CT scan (15). Accordingly, in our population we depicted GGO in all the included cases. In 50% of the cases GGO were the only lung abnormality, corresponding to the early stage of illness. In 30% of the cases consolidations were also noticed, implying a progressive or peak stage. Therefore, all our patients were in the initial phases of the disease. These results are similar to previously published findings of the recent Italian multicenter study of Albano et al (16). 

 FDG uptake is generally noted in several inflammatory and infective conditions of the lungs, with a large variability. This is due to the high glycolytic activity of cells involved in the inflammatory process. An increased uptake has been demonstrated also in cases of SARS-CoV-2 infection. It has been already demonstrated that SARS-CoV-2 causes inflammation by different mechanisms, involving macrophages, cytokines and T-cells. Specifically, SARS-CoV-2 enter cells binding the angiotensin-converting enzyme 2 (ACE2), part of the renin-angiotensin system (RAS), highly expressed on the apical side of lung epithelial cells in the alveolar space. By downregulating the RAS system and by other mechanisms, SARS-CoV-2 causes the inflammatory response (17). Being aware of the limited number of cases available, we found high variability in SUV_max_ values regardless the stage of disease, the type of CT patterns and the number of pulmonary involved lobes. Of note, we found higher SUV_max_ values in GGO pattern compared to consolidations. Our results are partially in contrast with the ones of Thornton et al., who found higher SUV_max_ values in more advanced stages of the disease characterized by lung consolidations (18). This may be explained by a lower prevalence of advanced stages with consolidations in our population. However, this variability implies a poor reliability of PET-CT in staging and monitoring the COVID-19 disease.

 Our findings are similar to the results reported in other previous studies: Qin et al [4] described 4 presumed cases with COVID-19 pneumonia. In all the patients at least two lobes showed FDG avidity of the GGOs, with SUV_max_ values ranging from 4.6 to 12.2. Differently from our population, in this study all the patients were symptomatic for fever or respiratory symptoms. Setti et al (6) reported a case series of 5 oncologic patients with pulmonary involvement of 2 or more lobes unrelated to cancer metastases. They found consolidation or GGOs with a wide range of FDG uptake (4.3-11.3). In this case series SARS-CoV-2 test has not been performed. GGO have many differential diagnosis (19), including first of all other bacterial and viral pneumonias, but also the interstitial lung diseases, pulmonary edema, ARDS and alveolar hemorrhage. The distribution of GGO, the medical history, patients’ symptoms and laboratory profile can settle the differential diagnosis. The most relevant differential diagnosis is certainly the adenocarcinoma that might present as GGO, either unifocal or multifocal. The recent advent of SARS-CoV-2 infection has added another potential issue in the differentiation of GGO. As a general rule, a lesion that disappears after medical treatment has a high probability to be a benign lesion, while a lesion growing over time suggests a malignant nature. According to literature, a GGO is more probable to be suggestive of a neoplastic condition when a solid component is present.

 Some studies have shown that the employment of PET-CT to distinguish between benign and malignant GGO nodules is inappropriate, especially in the case of pure GGO nodules (20). A short period of follow-up and an empiric therapy, eventually followed by lung biopsy, are considered the optimal method to distinguish between benign and malignant GGO lesions (21, 22). As proof of the inflammatory nature of lung disease in these patients we found a rapid evolution of the lung alterations over time: in two cases an improvement at imaging was observed after 1 months; in two other patients who underwent CECT about 7-10 days before PET scans, one (performed for a clinical trial) was completely negative; the other (performed for high D-dimer rates) showed initial GGO evolving in more compact consolidations in the subsequent PET-CT. In the current epidemiological context, with a high prevalence, GGO are more likely to be COVID-19-related, even when mixed to consolidations and a confirmation with a molecular test remains mandatory in doubtful cases (20). Otherwise, more studies will be required to assess any potential role of nuclear medicine, beside CT findings and the clinical profile of the patient, in the differentiation of GGO related to SARS-CoV-2 among other causes. 

 This retrospective study has some limitations. The first one is the small sample size, which limits the ability to apply a definite statistical evaluation and our findings to suggestions; however, as aforementioned, oncologic PET scanning in our Institution were significantly reduced by the severity of pandemic peak in the study period. Another limit is the lack of the molecular or serological confirmation in some of the cases, but the lung findings were similar and suggestive even in non-tested patients. 

 A possible perspective of these findings might be to compare in a high series of patients SUV_max_ values of SARS-CoV-2 related lung foci with other causes of pulmonary infections. 

## CONCLUSIONS

^18^F-FDG PET-CT in suspected COVID-19 pulmonary involvement of asymptomatic oncologic patients showed an increase in FDG uptake of GGO and consolidations, but with wide and overlapped range of SUV_max_ values. 

 Therefore, CT remains the mainstay for the assessment of lung involvement by SARS-CoV-2 infection, even in asymptomatic patients, recognized as potential source of infection spread.

## Ethics approval and consent to participate

 Institutional Review Board approval was obtained.

## Availability of data and materials

 The datasets generated and analyzed during the current study are not publicly available, but are available from the corresponding author on reasonable request.

## Competing interests

 The authors declare that they have no competing interests.
